# Operation on a Horse for Fistula

**Published:** 1896-11

**Authors:** 


Operation upon a Fistula of the Rectum. (Novotny, in *Thierärzt Centralblatt.*) A cavalry horse showed a small mass of matter in making evacuations. Novotny made an examination of the rectum and found a button-shaped excrescence the size of a hazelnut in the middle of the right wall of the rectum about 4 cm. from the anus. On pressing the excrescence a small mass of fetid matter mingled with blood presented. A probe could be pushed forward diagonally against the right branch of the intestine (junction of the cross-intestine bone) for 20 cm. without meeting any resistance in the whole canal. In three days a flat, painless swelling formed in the middle of the right rump. On forward movement of the foot about one-half litre of fetid matter exuded from the rectum. Not being able to find anything in regard to the treatment of such cases Novotny decided to open the swelling from the outside. At a depth of 10 to 13 cm. he found a hole the size of a man's head, from which one-fourth kilogram of dead tissue was removed. This hole communicated with a second, which opened near the transverse prolongations of the cross-bone, and extended toward the anus, terminating in the rectum. A drainage-tube was placed in the hole and through the fistula, and the wound was sprinkled with iodoform and cotton laid upon it. With the aid of a 3 per cent. creolin solution healing took place in eight weeks.—*Schweizer Archiv*, January and February, 1896.

